# Biphasic pulses enhance bleomycin efficacy in a spontaneous canine genital tumor model of chemoresistance: Sticker sarcoma

**DOI:** 10.1186/1756-9966-27-58

**Published:** 2008-11-03

**Authors:** Enrico P Spugnini, Ivan Dotsinsky, Nikolay Mudrov, Gennaro Citro, Alfredo D'Avino, Alfonso Baldi

**Affiliations:** 1 SAFU Department, Regina Elena Cancer Institute, Rome, Italy; 2 Centre of Biomedical Engineering "Ivan Daskalov", Sofia, Bulgaria; 3 Department of Biochemistry, section of Pathology, Second University of Naples, Naples, Italy

## Abstract

Sticker's sarcoma (also known as transmissible venereal tumor) is a horizontally transmitted neoplasm of the dog, that is passed with coitus. It is a locally aggressive tumor with a low tendency to metastatic spread. The most common locations are the genitals, the nose, the perianal area. Standard treatment consists with chemotherapy with vincristine, however other therapies such as, cryotherapy, immunotherapy or, in selected cases, radiation therapy, have been reported. In this article we describe the outcome of a small cohort of canine patients, with chemotherapy resistant transmissible venereal tumor (TVT), treated with bleomycin selectively driven by trains of biphasic pulses (electrochemotherapy). Three canine patients, with refractory TVT, entered the study and received two sessions of ECT under sedation. The pets had local injection of bleomycin at the concentration of 1.5 mg/ml and five minutes after the chemotherapy, trains of 8 biphasic electric pulses lasting 50 + 50 μs each, with 1 ms interpulse intervals, were delivered by means of modified caliper or, for difficult districts, through paired needle electrode. All the patients responded to the treatment and are still in remission at different times. Electrochemotherapy appears as a safe and efficacious modality for the treatment of TVT and warrants further investigations.

## Introduction

Canine Sticker's tumor is a coitus transmissible disease that is directly transferred from dog to dog through major histocompatibility complex barriers and by means of direct transfer of viable tumor cells from individual to individual [1]. The transfer is facilitated by the presence of mucosal lesions (like those occurring during mating), and can be transmitted by behaviors such as licking or sniffing [1]. Interestingly, the transmissible agent causing transmissible venereal tumor (TVT) is thought to be the tumor cell itself. Recent articles analyzed several genetic markers including major histocompatibility (MHC) genes, in Sticker's sarcomas and matched blood samples [2,3]. In each case, the tumor resulted genetically distinct from its host [2,3]. During progressive growth, TVT downmodulates MHC antigen expression and release inhibiting cytokines that impair the NK function, frequently resulting in host's inability to reject the tumor [4-6]. The treatment of election for these neoplasms is chemotherapy with vincristine however, cryotherapy, immunotherapy and, in selected cases, radiation therapy have been reported as well [1,7-10]. A recently published paper reports a response rate to vincristine of 31 out of 38 dogs, the non responders were treated with doxorubicin; at the completion of the chemotherapy cycles two dogs still had viable tumor cells [7].

Biphasic pulses electrochemotherapy (ECT) is a novel anticancer technique that has been recently investigated in oncology [11,12]. More recently, our group has investigated the feasibility and efficacy of ECT in companion animals carrying spontaneously occurring neoplasms, obtaining an high percentage of responses, many of which long lasting, even in neoplasms, such as lymphoma and soft tissue sarcomas, known to be non-responsive to bleomycin, the drug of election [13-29].

On the basis of such results, a study was performed to assess the potentials of this technique for the treatment or palliation of canine TVTs that were resistant to systemic chemotherapy due to the difficulties posed by treating tumors in the genital district.

## Materials and methods

### Patient selection

Privately owned veterinary patients with histopathologically confirmed localized TVT were selected for the study. Previous informed consent was obtained from the owners, according to the Italian law (116/92). In order to be enrolled, the patients had to have normal renal and hepatic function (normal serum blood urea nitrogen [BUN], creatinine and urine specific gravity as well as normal liver enzymes and bile acids). Moreover, patients had to be free of underlying life threatening diseases or other medical complications (i.e. diabetes mellitus).

**Figure 1:**
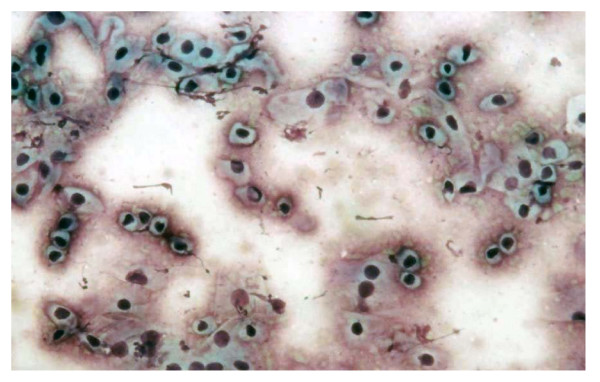
**A cytology slide of the neoplasm of one of the patients.** Note the numerous population of discrete round cells. (Diff Quick staining; magnification 100 ×).

Staging process included a thorough anamnesis, physical examination, complete blood cell count (CBC), serum biochemistry profile, iliac lymph node aspiration and cytological examination (if enlarged at ultrasonographic exam), thoracic radiographs (three projections), and tumor biopsy. In order to confirm the diagnoses, cytological examination of a biopsy (Figure 1), was performed by one of the authors, subsequently histological examination of the biopsies was performed following standard protocols, using Hematoxylin/Eosin and Hematoxylin/Van Gieson stainings by two of the authors.

All the patients had an ultrasonographic evaluation of the abdomen as well at the time of the enrollment in the study to rule out underlying disease. The characteristics of the enrolled patients are summarized in table 1.

**Table 1:** Individual data and response to electrochemotherapy (ECT) in three dogs with TVT

Signalment (age, sex,)	Tumor type and stage	Response (Months)	Outcome
4 yo, M, Hunting dog	T_2_N_0_M_0_	CR 48	Alive, CR
5 yo, M, Mixed Breed	T_3_N_0_M_0_	CR 34	Alive, CR
7 yo, M, German Shephard	T_1_N_0_M_0_	CR 28	Alive, CR

yo = years old; M = male CR = complete remission;

NB = age is given at the time of presentation;

### Treatment

Patients that entered in the study received two sessions of ECT one week apart under sedation with medetodimine and ketamine as per manufacturer's instruction. Briefly the tumor's bed and the margins for 0.5 cm in all directions were infiltrated with bleomycin at the concentration of 1.5 mg/ml. Five minutes after the infiltration, trains of 8 biphasic electric pulses lasting 50 + 50 μs each, with 1 ms interpulse intervals, were delivered by means of modified caliper electrodes. For particularly difficult districts, paired needle array electrodes were adopted.

Response to treatment and local toxicity were assessed prior to each therapy and two weeks after the second ECT. Criteria for tumor response to therapy are summarized in Table 2. Patients had monthly rechecks for the first six months and every third month thereafter. At that time, abdominal ultrasonographic exam was made to check for systemic spread. Toxicity was defined as disease processes that occurred secondary to changes of the tissues within the treatment field.

**Table 2:** Tumor response criteria

**Complete Remission **(CR) – the disappearance of all evidence of cancer in all sites for a defined period of time.
**Partial Remission **(PR) – the decrease in size of all tumors by 50% or greater as measured by the sum of the product of two diameters of each tumor for a defined period of time.
**Stable Disease **(SD) – the decrease of < 50% or an increase of < 25% in the sum of the product of two diameters for a defined period of time.
**Progressive Disease **(PD) – the increase of 25% or more in the sum of the product of two diameters for a defined period of time.

### Pulse generator

The "Chemopulse" is built up by a toroidal core transformer generating a roughly rectangular pulse which is split in two halves that are sequentially driven to obtain a biphasic pulse. The pulses are not singularly produced but are created in bursts of eight, thus reducing the treatment time and the overall patient morbidity. The equipment allows to choose among a broad range of voltages (from 450 to 2450 V) with sequential increases of 200 V and permits to regulate the number of pulses (from 1 to 16) and the duration of the pulses (50 to 100 μs). The standard setting is 8 pulses of 50+50 μs at 1300 V/cm. The pulse repetition frequency is 1 Hz while the frequency of burst repetition is 1 kHz, resulting in a total burst duration of 7.1 ms [11,13-29].

### Electrodes(14)

1) Modified monolateral compass electrode steel, bachelite, plastic with perforated metal plates. Dimensions (Length × Height × Width): 22 × 10 × 1 mm.

Vaccine type twin needle array electrode steel and plastic. Needle length: 20 mm; array diameter: 20 mm.

## Results

**Figure 2:**
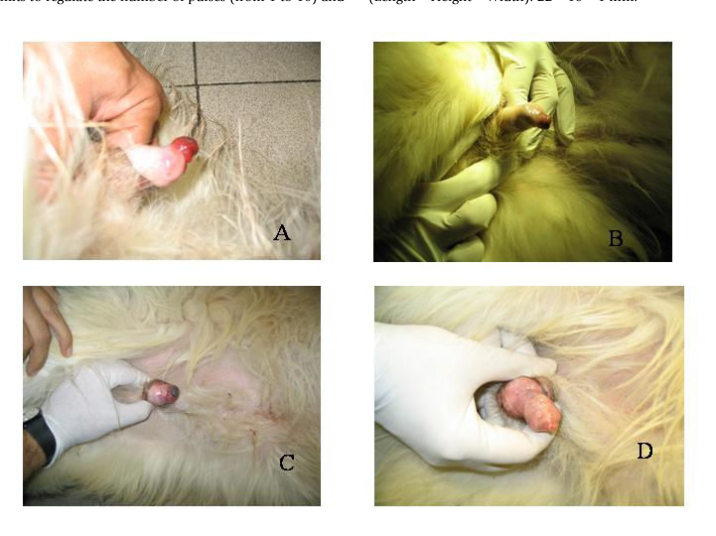
**A: Close-up image of a 4 year-old male Hunting dog, affected by a large TVT, at presentation.** B: the same patient after one session of ECT, note the tumor shrinkage. C: the same patient after the second session of ECT, the tumor has been replaced by scar tissue. D: the patient' genitals one month after the completion of the two cycles of electrochemotherapy.

Three dogs entered the study over a 6 years period; all the dogs were intact males, the ages were respectively 4, 5 and 7 years. Patients characteristics are summarized in Table 1. The fine needle aspiration of the lesions yielded a numerous population of discrete round cells (Figure 1) that had a round nucleus and a variable amount of cytoplasm. Anamnestically, the first dog had a partial remission after 6 doses of vincristine and two of doxorubicin, the second had a progressive disease after 4 doses of vincristine and one of doxorubicin, and the third was enrolled after failing after 4 doses of vincristine, due to a bad cardiac fraction shortening that prevented the use of anthracyclines. All the patients had a response to the first ECT session resulting in tumor shrinkage and decreased bleeding that evolved into a complete remission after the second ECT therapy. The complete responses lasted from 28 months to 48 months. Throughout the study, pulse-mediated bleomycin chemotherapy has been well tolerated by the enrolled patients, showing remarkable efficacy, while the only toxicity identified so far, is limited to mild swelling and erythema that subsided within 3–4 days after ECT in patient number 1. The therapy allowed the restoration of continence in two dogs and allowed a physiological urination in all the patients within 5 days from the first ECT. Furthermore, in all the patients, ECT has been instrumental to reduce the penile bleeding (Figure 2) thus, almost immediately, greatly improving the dogs' quality of life.

## Discussion

Electrochemotherapy is a novel technique for cancer treatment or palliation that showed high efficacy and low toxicity. Its ease of administration associated with its affordable cost that enables clinicians to treat again previously electroporated areas without side effects like cheloids, profuse bleeding, impaired wound healing like those observed in case of re-irradiation of tumors both in pets and humans [16,24,27], make it an appealing alternative to standard anticancer options. In the present study, electrochemotherapy, has proven to be a low toxicity therapy allowing local tumor control without significant local or systemic side effects. To the best of the authors' knowledge this is the first study on the adoption of ECT for the palliation/treatment of cytologically or histologically confirmed TVTs in a spontaneous canine genital tumor model. Despite the low number of enrolled patients, the long term responses obtained in all dogs with refractory disease are worth of mention. Further studies, investigating other tumor types involving the genitals, are needed to improve ECT control by exploring new protocols and drugs also in view of the possible translation of data to humans [24,27].

## Authors' contributions

EPS and GC performed the clinical trial, DI and NM helped with the equipment settings, DA and BA performed the histological analysis.

## Competing interests

The authors do not have competing interests.
